# Scalable Synthesis of Oxygen Vacancy-Rich Unsupported Iron Oxide for Efficient Thermocatalytic Conversion of Methane to Hydrogen and Carbon Nanomaterials

**DOI:** 10.3390/nano13172461

**Published:** 2023-08-31

**Authors:** Abdulrahman I. Alharthi, Talal F. Qahtan, Maged N. Shaddad, Mshari A. Alotaibi, Satam Alotibi, Amani M. Alansi

**Affiliations:** 1 Chemistry Department, College of Science and Humanities in Al-Kharj, Prince Sattam Bin Abdulaziz University, P.O. Box 83, Al-Kharj 11942, Saudi Arabia; m.shaddad@psau.edu.sa (M.N.S.); alosaimi@psau.edu.sa (M.A.A.); 2 Physics Department, College of Science and Humanities in Al-Kharj, Prince Sattam Bin Abdulaziz University, P.O. Box 83, Al-Kharj 11942, Saudi Arabia; t.qahtan@psau.edu.sa (T.F.Q.); sf.alotibi@psau.edu.sa (S.A.); 3 Chemistry Department, King Saud University, Riyadh 12372, Saudi Arabia

**Keywords:** unsupported α-Fe_2_O_3_ nanoparticles, hydrogen formation, methane decomposition, calcination process, oxygen vacancies, carbon nanomaterials

## Abstract

Thermocatalytic methane decomposition (TCMD) involving metal oxides is a more environmentally friendly and cost-effective strategy for scalable hydrogen fuel production compared to traditional methane steam reforming (MSR), as it requires less energy and produces fewer CO/CO_2_ emissions. However, the unsupported metal oxide catalysts (such as α-Fe_2_O_3_) that would be suited for this purpose exhibit poor performance in TCMD. To overcome this issue, a novel strategy was developed as a part of this work, whereby oxygen vacancies (OVs) were introduced into unsupported α-Fe_2_O_3_ nanoparticles (NPs). Systematic characterization of the obtained materials through analytical techniques demonstrated that mesoporous nanostructured unsupported α-Fe_2_O_3_ with abundant oxygen vacancies (OV-rich α-Fe_2_O_3_ NPs) could be obtained by direct thermal decomposition of ferric nitrate at different calcination temperatures (500, 700, 900, and 1100 °C) under ambient conditions. The thermocatalytic activity of the resulting OV-rich α-Fe_2_O_3_ NPs was assessed by evaluating the methane conversion, hydrogen formation rate, and amount of carbon deposited. The TCMD results revealed that 900 °C was the most optimal calcination temperature, as it led to the highest methane conversion (22.5%) and hydrogen formation rate (47.0 × 10^−5^ mol H_2_ g^−1^ min^−1^) after 480 min. This outstanding thermocatalytic performance of OV-rich α-Fe_2_O_3_ NPs is attributed to the presence of abundant OVs on their surfaces, thus providing effective active sites for methane decomposition. Moreover, the proposed strategy can be cost-effectively scaled up for industrial applications, whereby unsupported metal oxide NPs can be employed for energy-efficient thermocatalytic CH_4_ decomposition into hydrogen fuel and carbon nanomaterials.

## 1. Introduction

Owing to the growing demand for the abandonment of fossil fuels and the adoption of clean energy sources, academic research into applications involving hydrogen has increased considerably in recent years. The available evidence indicates that electricity can be efficiently obtained from hydrogen-based sources without any environmental pollution [1,2,3,4]. However, at present, hydrogen is typically attained through methane steam reforming (MSR) and partial natural gas oxidation, resulting in considerable CO_2_ emissions [5,6,7]. Thus, greener methane decomposition strategies are urgently required, as methane is superior to all other hydrocarbons in terms of the hydrogen/carbon ratio. Moreover, it is more abundant in nature than other fossil fuels and can be decomposed into H_2_ and C via a simple conversion process [8,9,10].

To overcome the aforementioned challenges inherent in currently used techniques, thermocatalytic methane decomposition (TCMD) based on metal oxide catalysts can be adopted, as it is cleaner and more cost-effective [8,11,12]. As it requires less energy and its CO/CO_2_ emission rate is lower than in MSR, it can be economically scaled up for practical industrial applications, including biomethane exploitation for carbon generation and storage [13,14]. In current TCMD processes, Ni-, Fe-, and Co-based catalysts are usually employed, along with Al_2_O_3_ and MgO or other metal oxides, which serve as supporting materials. Although Ni and Co particles are characterized by high activity in the 500−700 °C temperature range and their use results in a high graphitic carbon growth rate [5,6,12], Ni- and Co-based catalysts have some notable drawbacks, including high cost and toxicity. Moreover, at high temperatures—which are necessary for methane conversion at sufficiently high rates and for producing highly stable graphitic carbon—they undergo rapid deactivation [3]. In this respect, Fe-based catalysts are superior alternatives, as they maintain sufficiently high activity levels at temperatures up to 950 °C [15,16,17].

Therefore, Fe-based catalysts could be used for TCMD to produce H_2_ and carbon materials while benefiting from their non-toxic nature, wide availability, and high activity [18,19]. In particular, hematite (α-Fe_2_O_3_) can be utilized for this purpose, as it is the most thermodynamically stable iron oxide under ambient conditions and is relatively inexpensive, readily available, and environmentally safe. However, to facilitate the separation and recovery of catalysts and carbon products, magnetic unsupported solid metal catalysts are usually used; however, unsupported α-Fe_2_O_3_ catalysts currently have poor methane decomposition performance. To overcome this issue, various strategies have been proposed, including morphological control [20,21], element doping [22], heterojunction construction [22,23], and surface modification [24,25]. In this context, surface defects such as oxygen vacancies have emerged as a particularly promising approach, given that defect engineering (e.g., by creating oxygen vacancies in metal oxides) is widely used to tune catalyst performance [26,27]. Extant research shows that OVs can be employed to modulate the geometric configuration, electronic structure, and physicochemical properties of metal oxides. Moreover, oxygen-defective materials have been shown to serve as effective initiators for the adsorption of molecules in a wide range of surface reactions [28,29].

In practical applications, methane decomposition catalysts for hydrogen production are generally synthesized and/or prepared through impregnation, co-precipitation, and sol-gel processes. However, when a two-step preparation is conducted, catalyst morphology cannot be adequately controlled, and its structure changes with the addition of metals, thus affecting the particle distribution and size. Therefore, in this work, a novel method for the development of unsupported α-Fe_2_O_3_ nanoparticles (NPs) for thermocatalytic methane decomposition without the use of any supporting agents is proposed. Specifically, oxygen vacancies are introduced into unsupported α-Fe_2_O_3_ NPs, making them suitable for TCMD. The catalyst is prepared via the calcination method at temperatures (500, 700, 900, and 1100 °C) under ambient conditions. During the calcination process, OVs are created on the surface of the unsupported α-Fe_2_O_3_ NPs. Thus, this one-step, highly efficient, and controllable method is highly cost-effective and scalable. In the sections that follow, we report on the preparation, characterization, and catalytic performance of mesoporous nanostructured unsupported α-Fe_2_O_3_ with abundant oxygen vacancies (OV-rich α-Fe_2_O_3_ NPs), which were used for efficient thermocatalytic methane conversion to hydrogen and carbon nanomaterials.

## 2. Experimental

### 2.1. Preparation of Unsupported OV-Rich α-Fe_2_O_3_ NPs

**Figure 1:**
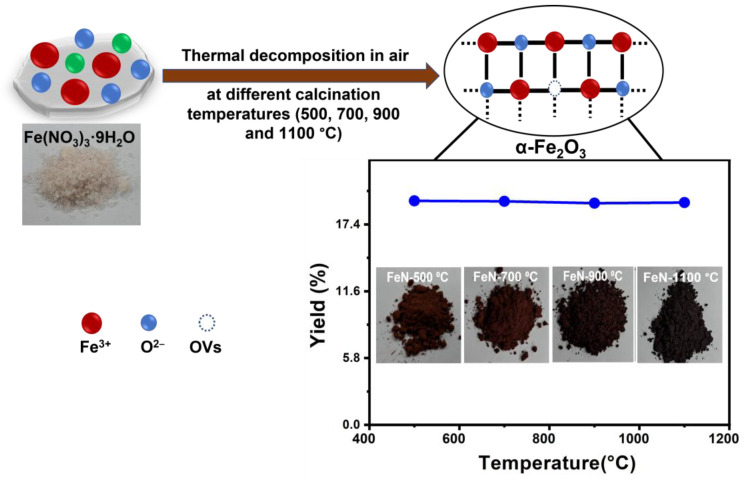
The preparation method of the obtained OV-rich α-Fe_2_O_3_ NPs at different calcination temperatures for 5 h at ambient conditions.

OV-rich α-Fe_2_O_3_ NPs were prepared via thermal decomposition of ferric nitrate nonahydrate Fe(NO_3_)_3_.9H_2_O (which served as a metal precursor) at different temperatures. In the typical procedure, 5 g of Fe(NO_3_)_3_.9H_2_O (Purchased from LOBA CHEMIE PVT.LTD., Mumbai, India, purity of 98%) was calcinated at 500, 700, 900, and 1100 °C in ambient air for 5 h (5 h) to obtain samples denoted as FeN-500 °C, FeN-700 °C, FeN-900 °C, and FeN-1100 °C, respectively as indicated in Figure 1. Next, unsupported OV-rich α-Fe_2_O_3_ NPs were obtained by cooling the as-prepared samples to room temperature.

### 2.2. Characterization Techniques

For crystalline phase identification, the obtained fresh OV-rich α-Fe_2_O_3_ NPs and spent samples collected after use for TCMD were studied via X-ray diffraction (XRD) analysis over the 2θ = 10−70° scanning range, using the X-ray diffractometer (Rigaku International, Tokyo, Japan), Cu Kα radiation (λ = 1.5406 A). The surface morphology of the obtained fresh OV-rich α-Fe_2_O_3_ NPs and spent samples were observed using a scanning electron microscope (FESEM, QUANTA 250 FEI; Hillsboro, OR, USA), while the textural properties of the fresh samples, including surface area, pore volume, and pore radius, were measured using N_2_ physisorption isotherms using a Quantachrome Autosorb IQ model ASIQA3V600000–6 instrument. X-ray photoelectron spectroscopy (XPS) was further employed to investigate the near-surface chemical composition and oxidation states of the studied materials. A Thermo K Alpha spectrometer operating at 1486.6 eV with a 400 μm spot size and charge correction was utilized for this purpose. As an internal standard, all binding energy estimates were calibrated to the C 1*s* energy (284.5 eV). In addition, thermogravimetric analyses (TGA) were carried out in ambient air using a Netzsch proteus 70 instrument at 10 °C/min increments within the 25–1000 °C temperature range. Collected TGA data demonstrated typical mass loss with regard to temperature, and their corresponding first derivative thermogravimetric (DTG) graphs provided information on mass loss rate versus temperature.

### 2.3. Thermocatalytic Activity Experiments

The activity of unsupported nanostructured OV-rich α-Fe_2_O_3_ NPs was investigated in a quartz tubular fixed-bed reactor with a 1.5 cm internal diameter and 50 cm total length. For this purpose, 0.5 g of the previously prepared fresh catalyst sample was placed in the center of the tube between the ceramic fiber plugs preheated to 800 °C. Next, a 3:1 mixture of CH_4_ and N_2_ feed gas (supplied by AHG at 99.99% purity) was introduced into the reactor at a 20 mL/min flow rate, which was controlled by a mass gas flowmeter (MKS PR4000B). During data analysis, the nitrogen present in the mixture was used as the internal standard. The OV-rich α-Fe_2_O_3_ NPs were not subjected to any pretreatment because methane is considered a reductant. Reaction products were examined using online Gas Chromatography, on an Agilent GC Model 7890B equipped with 19043 Restek Micro packed GC Columns (Shin carbon ST 80/100 2 M, 0.53 mm), while a thermal conductivity detector was utilized to analyze the output gas and determine the hydrogen, nitrogen, and unreacted methane content.

## 3. Results and Discussion

### 3.1. Characterization of Fresh Samples

The synthesis of OV-rich α-Fe_2_O_3_ NPs through the calcination of pure ferric nitrate nonahydrate (Fe(NO_3_)_3_·9H_2_O) at different temperatures (500, 700, 900, and 1100 °C) under ambient conditions is an important aspect of this study, as it allows for the tailoring of the properties of the resulting NPs to optimize their catalytic performance in thermocatalytic methane decomposition.

Interestingly, we observed that the mass of the obtained OV-rich α-Fe_2_O_3_ NPs remained constant at different calcination temperatures, as demonstrated in the inset of Figure 1. This finding suggests that the yield of the calcination process is independent of the calcination temperature.

This observation is crucial for the scalability of the synthesis process, as it indicates that the amount of OV-rich α-Fe_2_O_3_ NPs that can be obtained is not limited by the calcination temperature. Therefore, this synthesis method can be easily scaled up to produce large quantities of OV-rich α-Fe_2_O_3_ NPs for industrial applications, such as energy-efficient thermocatalytic methane decomposition into hydrogen fuel and carbon nanomaterials.

**Figure 2:**
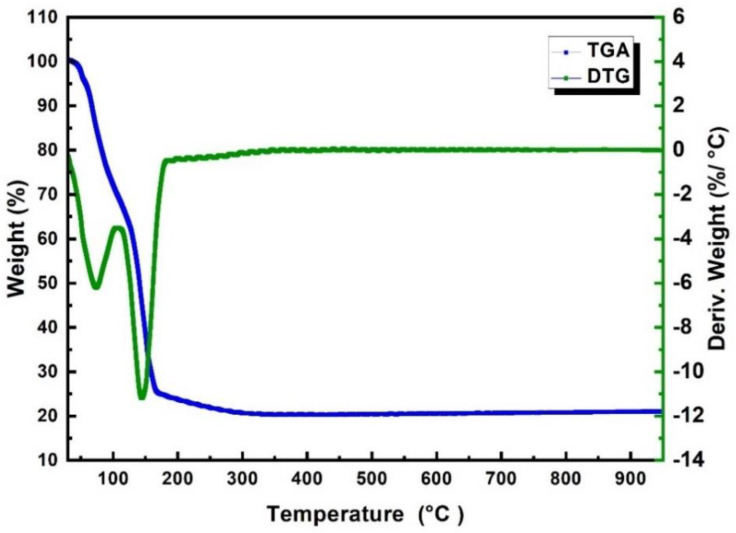
TGA/DTG curves for the Fe(NO_3_)_3_·9H_2_O in the presence of air.

Figure 2 depicts the TGA-DTG curves of pure Fe(NO_3_)_3_·9H_2_O under ambient conditions, demonstrating two distinct mass loss regions. These regions indicate that the decomposition of Fe(NO_3_)_3_·9H_2_O salt into Fe_2_O_3_ occurs in two main steps. The first step occurring between 75 and 150 °C could be attributed to the removal of the crystalline water bound to the salt and the decomposition of the nitrates within the salt, which corresponds to the formation of ferric oxyhydroxide (FeOOH). The second step, occurring between 150 and 300 °C, indicates the formation of Fe_2_O_3_ [30].

Furthermore, a slight decrease observed before the temperature of 75 °C could potentially be attributed to the loss of adsorbed water weight. The loss of weight corresponding to the removal of adsorbed water from the precursor salt typically begins between 45 and 70 °C, which agrees with our results in Figure 2. It is worth noting that the TGA-DTG analysis reveals that calcination temperatures of around 500 °C and above are effective in removing the nitrate and hydrate groups associated with the ferrous metal. This observation is consistent with the negligible change in the weight of the resulting Fe_2_O_3_ when increasing the calcination temperature from 500 °C to 1100 °C at ambient conditions, as shown in Figure 1.

**Figure 3:**
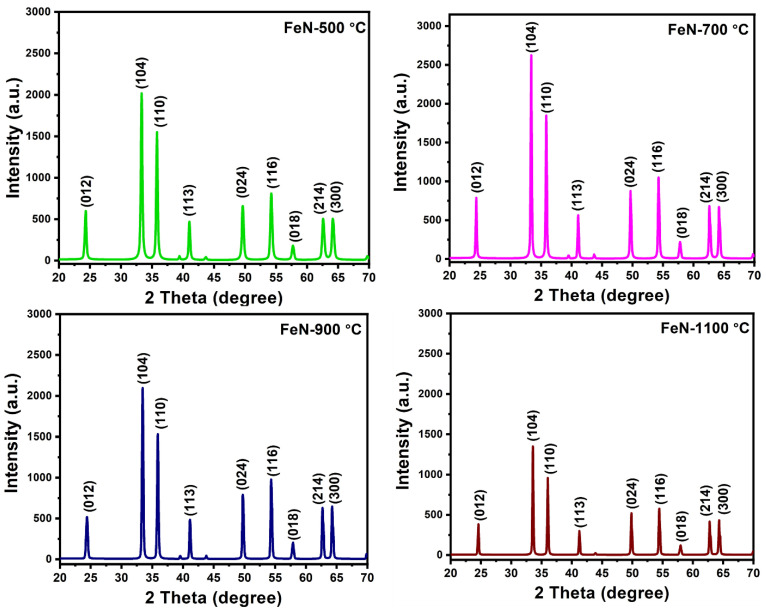
XRD patterns of the obtained OV-rich α-Fe_2_O_3_ NPs at different calcination temperatures for 5 h at ambient conditions.

XRD was employed to examine the crystalline structure and determine the phase purity of the obtained OV-rich α-Fe_2_O_3_ NPs at different calcination temperatures, and the obtained diffraction patterns are shown in Figure 3.

The diffraction peaks that can be observed in the XRD patterns are centered at the 2θ = 24.3°, 33.4°, 35.8°, 41.1°, 49.7°, 54.5°, 57.9°, 62.4° and 64.4°, and thus correspond to the (012), (104), (110), (113), (024), (116), (018), (214), and (300) diffraction planes of hematite α-Fe_2_O_3_ (PDF Number 33-0664) [31]. The absence of any secondary phases confirms that Fe(NO_3_)_3_.9H_2_O has completely decomposed to the α-Fe_2_O_3_ hematite phase. Figure 3 demonstrates an increase in the intensity of the diffraction peaks upon increasing the calcination temperature to 700 °C, providing the highest degree of crystallinity for the obtained hematite, α-Fe_2_O_3_. The calcination process at 900 and 1100 °C results in a slight decrease in crystallinity of the formed hematite phase, which is attributed to the sintering of the nanoparticles at these temperatures, which may lead to the formation of defects [32].

**Figure 4:**
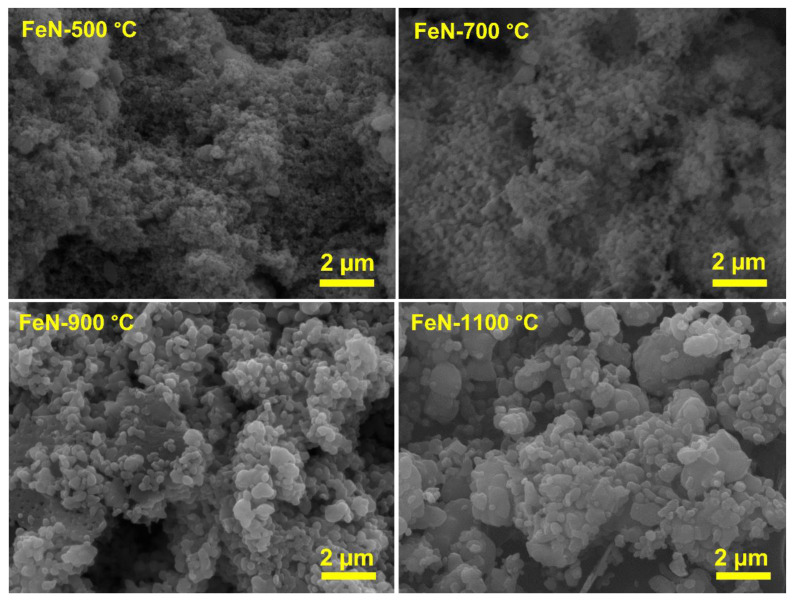
SEM images of the obtained OV-rich α-Fe_2_O_3_ NPs at different calcination temperatures for 5 h at ambient conditions as indicated.

Scanning electron microscopy (SEM) was employed to examine the morphological features of the obtained OV-rich α-Fe_2_O_3_ NPs at different calcination temperatures, and the results are depicted in Figure 4. From Figure 4, it can be seen that the particle size changes gradually with increasing calcination temperatures from 500 to 1100 °C. Although the particle size increases slightly at 700 °C, it remains small and randomly interconnected on a cotton-like surface. The effect of the calcination temperature between 900 and 1100 °C is clearer, as the particles are formed from quasi-spherical shapes and undefined shapes with different sizes, and their sizes increased with the increase in calcination temperatures from 900 to 1100 °C. It is known that when the calcination temperature rises, the particle size becomes larger due to particle growth and/or agglomeration [12].

**Figure 5:**
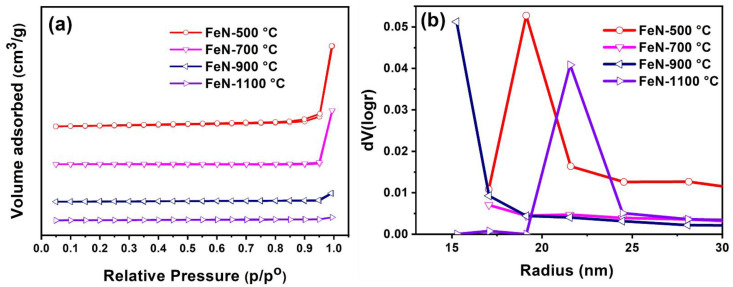
(**a**) Nitrogen adsorption/desorption isotherms and (**b**) BJH pore size distribution of the obtained OV-rich α-Fe_2_O_3_ NPs at different calcination temperatures for 5 h at ambient conditions.

The textural properties of the OV-rich α-Fe_2_O_3_ NPs were measured utilizing N_2_ adsorption-desorption isotherms and Brunauer-Emmett-Teller (BET)/Barrett-Joyner-Halenda (BJH) analysis, and the findings are presented in Table 1 and Figure 5. As can be seen from the data reported in Table 1, calcination temperature significantly affected the BET surface parameters of the studied materials, whereby the BET surface area declined from 16.70 to 3.90 m^2^/g as the calcination temperature increased from 500 to 1100 °C.

This trend could be attributed to particle agglomeration associated with elevated temperatures. Figure 5a displays the N_2_ adsorption−desorption isotherms of the obtained OV-rich α-Fe_2_O_3_ NPs at different calcination temperatures and shows that they are of type IV with H3 hysteresis loop, which is typical of mesoporous hematite α-Fe_2_O_3_ [33]. Furthermore, as the hysteresis loop areas decline with increasing calcination temperature, this finding further confirms that calcination may induce the collapse of some porous materials [34]. Usually, H3-type hysteresis is produced by solids with aggregate or agglomerate particles of non-uniform size and/or shape [35]. The isotherms depicted in Figure 5b reveal the mesoporous nature of OV-rich α-Fe_2_O_3_ NPs based on the pore size distribution, which remained in the 19−22 nm range.

Thus, from the SEM images (Figure 4), it is concluded that the particle size increased with the increase in the calcination temperature due to the sintering and/or agglomeration of the particles. This observation is consistent with the results of the surface area analysis (Table 1), as it decreased with the increase in calcination temperature.

**Figure 6:**
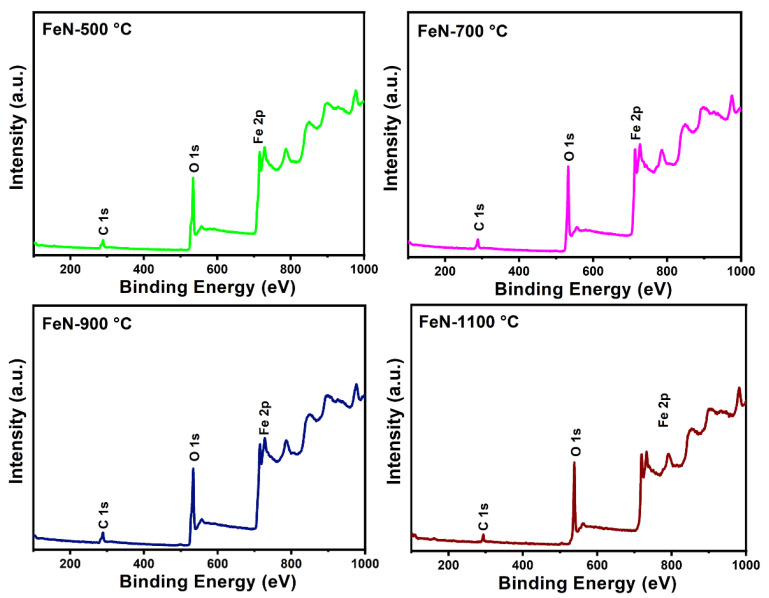
Survey XPS spectra of the obtained OV-rich α-Fe_2_O_3_ NPs at different calcination temperatures for 5 h at ambient conditions.

**Figure 7:**
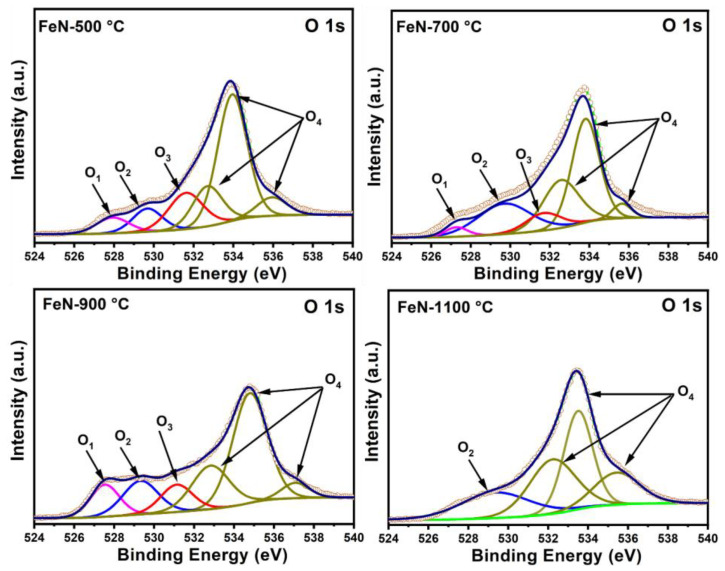
The high-resolution XPS spectra of O 1s for the obtained OV-rich α-Fe_2_O_3_ NPs at different calcination temperatures for 5 h at ambient conditions.

The chemical composition and elemental states of the obtained OV-rich α-Fe_2_O_3_ NPs at different calcination temperatures were probed by X-ray photoelectron spectroscopy (XPS), and the findings are shown in Figure 6. The XPS survey spectra of the examined OV-rich α-Fe_2_O_3_ NPs depicted in Figure 6 confirm that Fe, O, and C are the only constituents, whereby the presence of carbon is mainly attributed to its absorption from the atmosphere before XPS measurements. On the other hand, the oxygen peaks indicate successful α-Fe_2_O_3_ formation. The O 1s spectra of the OV-rich α-Fe_2_O_3_ NPs samples presented in Figure 7 confirm the presence of four chemical states of the surface oxygen species. The main peak located at around 529.5 eV can be ascribed to the lattice oxygen of α-Fe_2_O_3_ (Fe^3+^–O, labeled as O_2_), while the signal at the 531.8 eV binding energy corresponds to the hydroxyl group (–OH, labeled O_3_). Interestingly, the peak resulting from the hydroxyl group shifts towards lower energy for the sample that was calcined at 900 °C, which indicates these samples are oxygen-deficient. Thus, the peak O_3_ at around 531.1 in the FeN-900 °C sample is attributed to the low-coordinated oxygen species adsorbed onto the oxygen vacancies, likely arising from the formation of oxygen vacancies on the NP’s surface. On the other hand, the peaks that appear in the 533–536 eV energy range (labeled as O_4_) may be related to the surface-adsorbed H_2_O molecules and carbonate species [27,29,36]. Finally, the peak (O_1_) that appeared at 527.6 eV in FeN-500 °C, FeN-700 °C, and FeN-900 °C samples is attributed to the fact that some of the Fe^3+^ on the surface can be changed to a low oxidation state such as Fe^2+^ which is usually associated with the formation of OVs. The OVs are expected to play an important role in the adsorption process, as these vacancy sites can act as initiators for the adsorption of molecules in many surface reactions (i.e., many species such as H_2_O and CO_2_ will adsorb on the OVs from ambient air).

As defect states (such as OVs) are known to appear on the surface of metal oxides at high temperatures, it is clear from Figure 7 that the number of defects increases with increasing temperature from FeN-500 °C-FeN-900 °C. Moreover, the O_2_ peak ascribed to the lattice oxygen in the FeN-900 °C sample is shifted to lower bending energy compared to the other samples, which implies the oxygen vacancies are present in high concentration in this sample.

**Figure 8:**
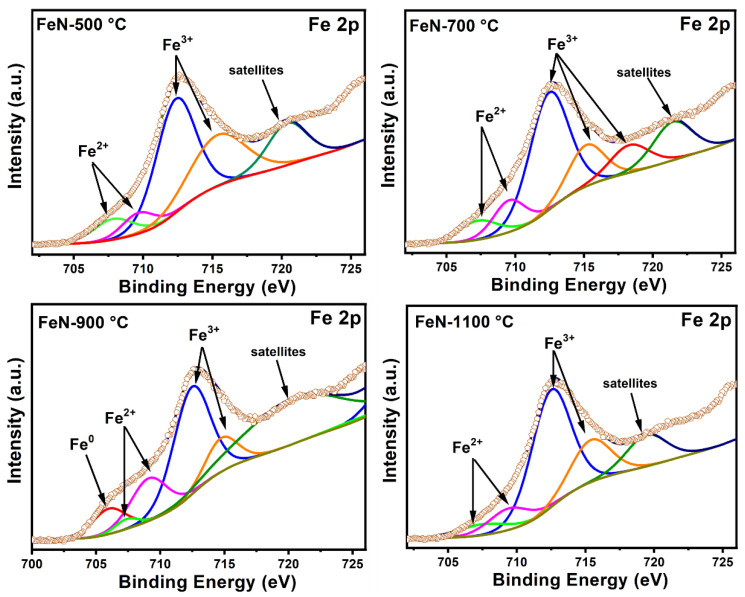
The high-resolution XPS spectra of Fe2p for the obtained OV-rich α-Fe_2_O_3_ NPs at different calcination temperatures for 5 h at ambient conditions.

Figure 8 illustrates the Fe 2p_3/2_ XPS spectra of the OV-rich α-Fe_2_O_3_ NPs obtained in this study. The spectra exhibit five peaks, with two peaks located at 707.6 and 709.7 eV attributed to Fe^2+^ and the peaks at 712.6 and ~715.4 eV corresponding to the oxidation state of Fe^3+^. Additionally, a peak at ~720.6 eV can be assigned to the satellite peak, as suggested by previous studies [29,37,38]. The Fe^2+^/Fe^3+^ ratio was calculated to be 0.20, 0.29, 0.36, and 0.22 for samples FeN-500 °C, FeN-700 °C, FeN-900 °C, and FeN-1100 °C, respectively, indicating the formation of OVs.

Notably, an additional small peak appeared at 706.2 eV in the FeN-900 °C sample, which is attributed to Fe^0^, suggesting that some Fe^3+^ was reduced into Fe^0^, a phenomenon commonly associated with the formation of OVs. The absence of this peak in other samples suggests that the FeN-900 °C sample may contain a higher concentration of OVs. OVs may accelerate the iron oxide reduction process and lead to the formation of more active sites, thereby enhancing catalytic activity.

### 3.2. Thermocatalytic Activity of the Obtained OV-Rich α-Fe_2_O_3_ NPs

**Figure 9:**
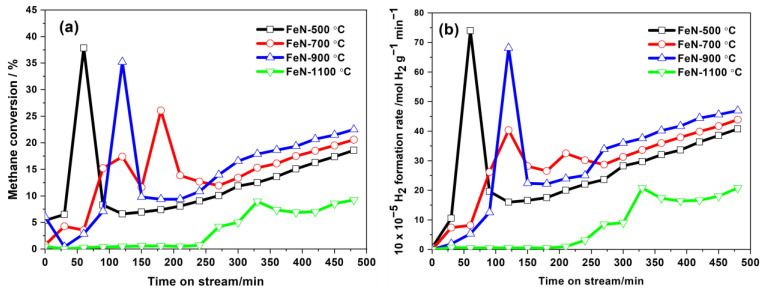
(**a**) Methane conversion percentage (%) and (**b**) Hydrogen formation rate as a function of time on stream (min) at 800 °C, 20 mL/min feed gas flow rate, and 0.5 g catalyst mass of the obtained OV-rich α-Fe_2_O_3_ NPs at different calcination temperatures for 5 h at ambient conditions. WHSV = 2.4 [L(g_cat_.h)].

The obtained OV-rich α-Fe_2_O_3_ NPs at different calcination temperatures were used as catalysts for the TCMD reaction. Their thermocatalytic activities were investigated at a fixed operating temperature of 800 °C and a gas flow rate of (20 mL/min). It should be noted that these materials did not undergo pre-reduction treatment before the reaction. Figure 9a,b show the methane conversion percentage (%) and hydrogen formation rate as a function of time on stream (TOS). From Figure 9a,b, it is clear that the prepared OV-rich α-Fe_2_O_3_ NPs at 1100 °C showed the lowest activity, where the highest methane conversions achieved were 9.20%, whereas the highest hydrogen formation rate was 20.80 × 10^−5^ mol H_2_ g^−1^ min^−1^. In contrast, the prepared OV-rich α-Fe_2_O_3_ NPs at 500, 700, and 900 °C displayed higher activity, which can be observed in the initial phase of the reaction, where the maximum methane conversions reached 37.8, 26.1, and 35.2%, respectively, while the maximum hydrogen formation rate was 74.0 × 10^−5^, 40.3 × 10^−5^ and 68.2 × 10^−5^ mol H_2_ g^−1^ min^−1^. It appears that these samples have a partially reducible component that forms active sites, which was followed by a rapid carbon deposit on them, subsequently suppressing their performance. Moreover, after 200 min of TOS, it is noted that the samples of OV-rich α-Fe_2_O_3_ NPs showed different activity as there was a gradual increase in their activity, and the order of performance was as follows: the prepared OV-rich α-Fe_2_O_3_ NPs at 900 °C > OV-rich α-Fe_2_O_3_ NPs at 700 °C > OV-rich α-Fe_2_O_3_ NPs at 500 °C > OV-rich α-Fe_2_O_3_ NPs at 1100 °C, and the maximum methane conversions achieved were 22.5, 20.6, 18.6, and 9.3%, respectively, whereas the maximum hydrogen formation rate was 47.0 × 10^−5^, 44.0 × 10^−5^, 40.7 × 10^−5^ and 20.83 mol H_2_ g^−1^ min^−1^, respectively.

Although these materials have the same phase of α-Fe_2_O_3_, the reaction was carried out under the same conditions, and the amount of catalyst and its iron content were constant, there is a variation in their activities. It appears that the surface area did not play a role in the activity of these materials, as increasing the calcination temperature led to an increase in the particle size and thus a decrease in the surface area, as seen in SEM images in Figure 4, and a sharp decrease in its surface area as shown in Table 1.

In an attempt to find the reasons for these disparate activities, it would appear to be due to the presence of defects in the iron oxide composition that arose from the effect of calcination temperatures. The results of XPS analysis (Figure 7 and Figure 8) showed that there are OVs on the α-Fe_2_O_3_ NPs surface, which may have played a significant role in the activity of these materials. It is noteworthy that the FeN-500 °C sample at the beginning of the reaction showed a high methane conversion percentage and hydrogen formation rate compared to the FeN-900 °C sample. However, the high activity of FeN-900 °C at the end of the TCMD reaction may be due to the presence of a high concentration of OVs on its surface, as observed in the XPS results in Figure 7 and Figure 8. The OVs on the α-Fe_2_O_3_ NPs surface play an important role in accelerating the iron oxide reduction process compared to those that contain fewer vacancies, which lead to the formation of more active sites during the TCMD, which act as effective active sites for the adsorption of methane. On the other hand, the low catalytic activity of the FeN-1100 °C catalysts can be attributed to the low concentration of the OVs, as is clear from the XPS results of these catalysts, which made them difficult to reduce during the reaction run period as these catalysts were not subjected to a prior reduction process [39].

It is worth mentioning that the activity of the prepared unsupported α-Fe_2_O_3_ catalysts by our scalable and cost-effective strategy is comparable with previously reported Fe-based catalysts for TCMD, as presented in Table 2, where WHSV is weight hourly space velocity.

### 3.3. Characterization of Spent Catalysts after the TCMD

**Figure 10:**
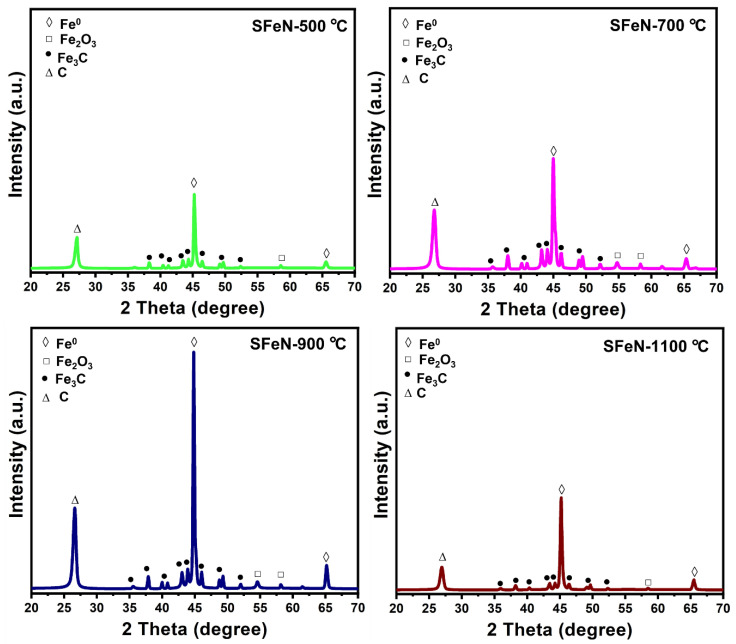
XRD patterns of spent catalysts obtained after TCMD.

Figure 10 shows the XRD patterns produced by spent catalysts obtained after methane decomposition over OV-rich α-Fe_2_O_3_ catalysts at 800 °C, revealing that the previously observed XRD peaks in Figure 3 have disappeared and OV-rich α-Fe_2_O_3_ catalysts have almost lost their original structure. It is worth noting that the spent catalysts are denoted as SFeN-500 °C, SFeN-700 °C, SFeN-900 °C, and SFeN-1100 °C. From Figure 10, it can be seen that the phase of OV-rich α-Fe_2_O_3_ catalysts has changed to Fe^0^, Fe_3_C, Fe_2_O_3,_ and graphite. Two clear peaks can be seen at 2θ = 26° and 2θ = 45° suggesting the formation of graphite carbon and metallic iron, respectively.

Furthermore, the presence of iron carbide (Fe_3_C, albeit in small amounts) is confirmed by the peak situated in the 2θ = 38–50° range, and it was suggested that Fe_3_C species have a catalytic activity for methane decomposition [44,45]. In addition, the appearance of very small peaks of Fe_2_O_3_ phases indicates that the catalysts were not completely reduced due to the carbon deposited on iron, explaining the continuous TCMD up to 480 min as observed in Figure 9 [46]. Generally, it can be suggested that the existence of metallic Fe and Fe_3_C phases in the catalyst component plays a role in the activity of the catalyst [46]. Interestingly, the diffraction peaks in the SFeN-900 °C catalyst showed higher intensity compared to other spent catalysts, suggesting its improved crystallinity degree.

**Figure 11:**
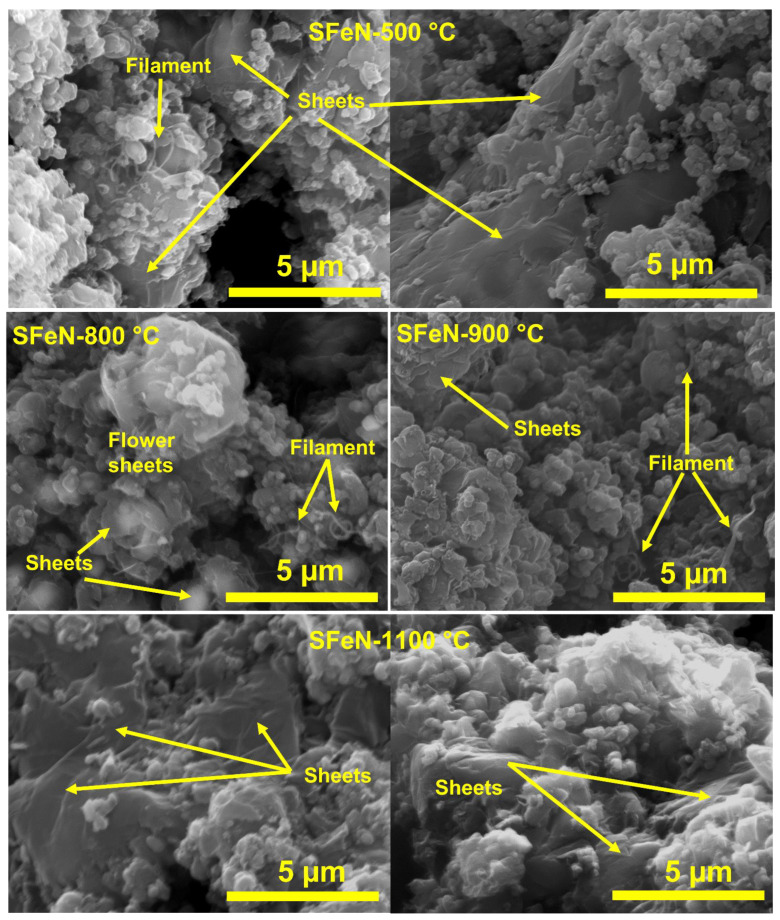
Scanning electron microscope images of spent catalysts obtained after TCMD.

The morphological characteristics of the deposited nanocarbon in spent catalysts were examined by performing SEM analyses, and the images are shown in Figure 11. The SEM images of all the spent catalysts, as a result of TCMD, demonstrated different morphologies of deposited nanocarbon. A negligible amount of carbon nanofilaments in the catalysts was produced, along with multilayered graphene or graphite nanosheets encapsulating the catalyst. It was reported that the scarcity of the appearance of filamentous carbon in the catalysts is attributed to the fact that unsupported metal oxide catalysts are unable to form filamentous carbon during the TCMD. This is due to the catalyst particles undergoing rapid accumulation with each other and forming large particles as a result of harsh reaction conditions [47]. While the appearance of many graphene sheets may be attributed to the high diffusion coefficient of carbon atoms through metallic Fe at 800 °C, which may lower the formation of metal-encapsulated carbon nano-chunks and further expedite the formation of graphene sheets [44], furthermore, it has been observed that irregularly shaped carbon nano-chunks have appeared in abundance in the SFeN-700 °C and SFeN-900 °C samples.

**Figure 12:**
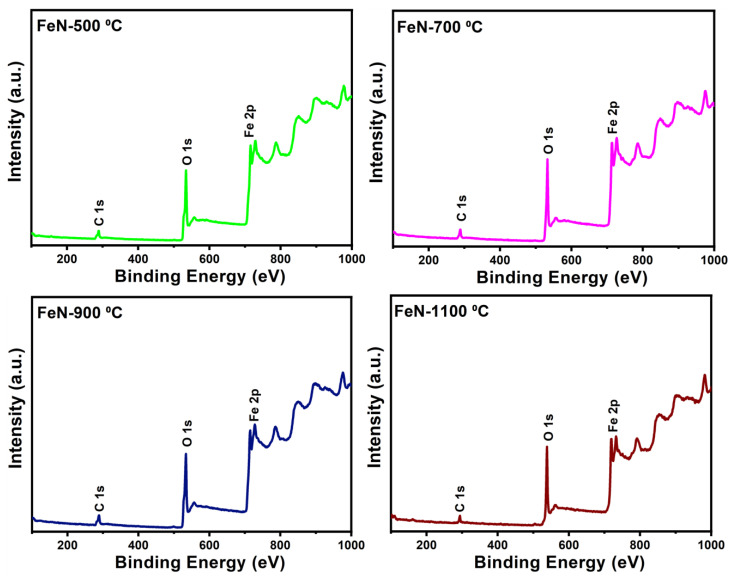
Survey XPS spectra of the spent catalysts obtained after TCMD.

**Figure 13:**
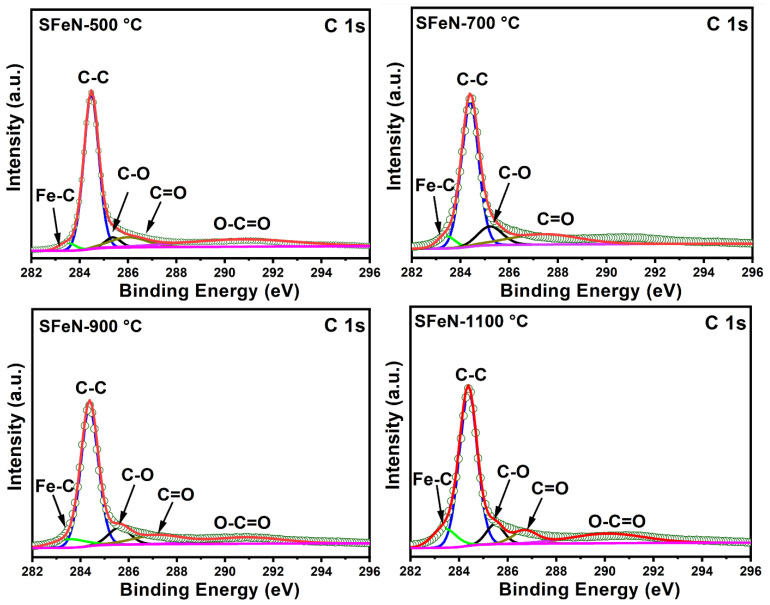
The high-resolution XPS spectra of C1s for the spent catalysts obtained after TCMD.

The XPS spectra of the spent catalysts shown in Figure 12 and Figure 13 indicate that all spent catalysts are rich in carbon. Moreover, a weak O 1s peak can be seen in the survey spectrum shown in Figure 12 while the Fe 2p peak is absent, suggesting that the α-Fe_2_O_3_ reduction, as well as carbon deposition, highlights the successful TCMD, which confirms XRD and SEM findings.

The high-resolution C 1s XPS spectra of the spent catalysts shown in Figure 13 can be deconvoluted into five peaks located at 283.3 eV, 284.4 eV, 285.2–286.0 eV, 286.8–288.0 eV, and ~291 eV, which can be indexed to carbide carbon (Fe–C), graphitic carbon (C–C, C–H), C–O bonds, C=O bonds, and O–C=O bonds, respectively, demonstrating the existence of the graphitic carbon [35,48]. Because Fe has a much higher atomic sensitivity factor compared to C and O, the absence of Fe peaks indicates that α-Fe_2_O_3_ was uniformly coated by thin carbon sheets. Furthermore, the presence of Fe-C agrees with the XRD results of spent catalysts and implies the formation of Fe_3_C, which has catalytic activity for methane decomposition.

**Figure 14:**
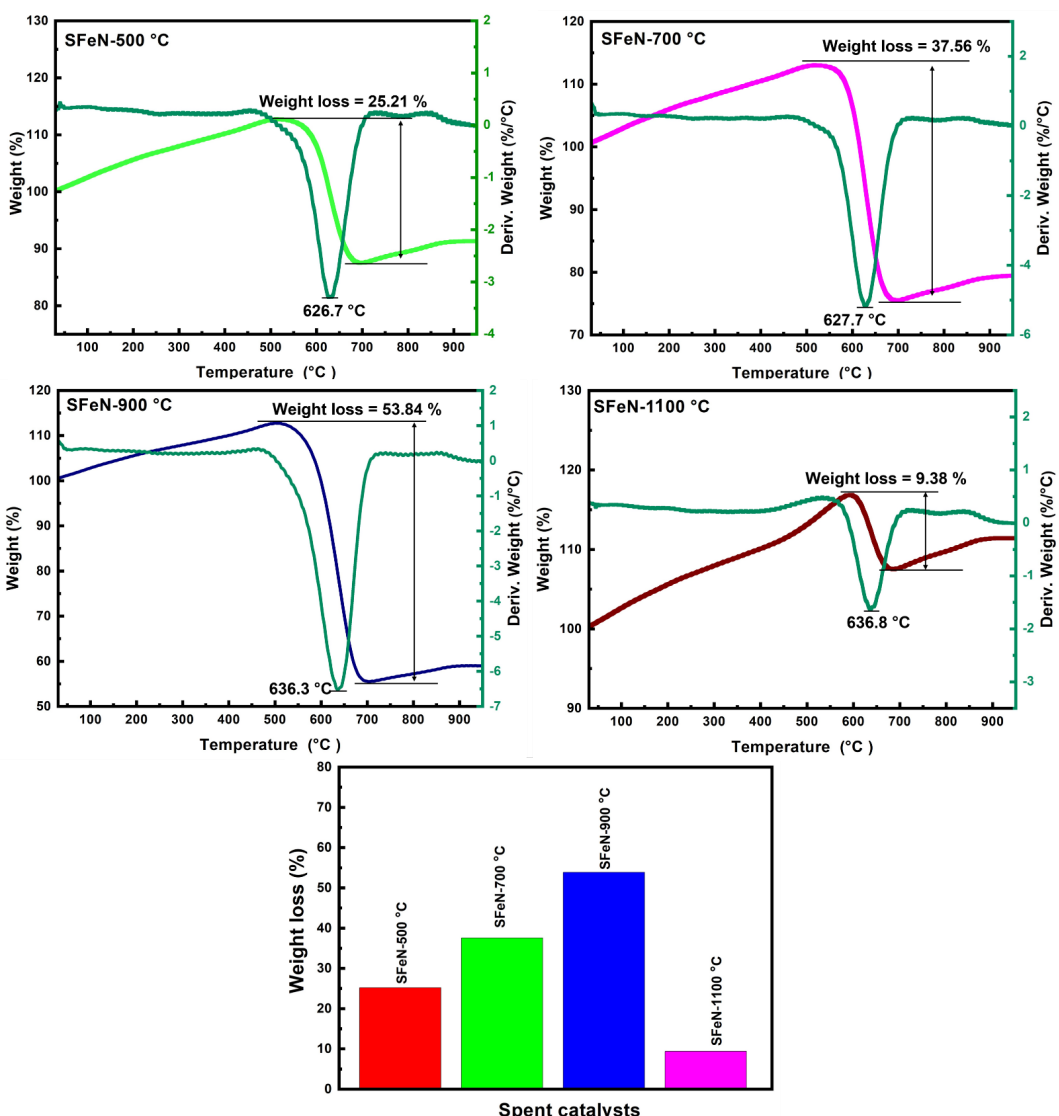
Typical TGA/DTA curves, as well as the loss of weight on the TGA curves of spent catalysts obtained after TCMD.

Figure 14 illustrates the plot of the TGA-DTG curves for the spent catalysts after TCMD. It depicts the weight loss of the spent catalysts when exposed to high temperatures in the presence of air.

Irrespective of the spent catalyst type, there is an initial increase in weight up to 500 °C, which may be related to the oxidation of the metallic iron. As temperatures increase beyond 500 to 700 °C, the weight loss in spent catalysts is remarkably different due to the oxidation of carbon nanomaterials.

It is observed from the TGA curves that the weight loss takes place in the range of 500–700 °C due to the combustion of graphitic carbon [43], which increases from SFeN-500 to SFeN-900 °C spent catalysts, as indicated in Figure 14. The difference in weight loss among the spent catalysts reflects the catalytic activity of the fresh catalysts; low catalytic activity shows less weight loss, and vice versa. These results agree with the methane conversion and hydrogen formation rate results, which indicated that the OV-rich α-Fe_2_O_3_ NPs prepared at 900 °C have the highest methane conversion (22.5%) and hydrogen formation rate (47.0 × 10^−5^ mol H_2_ g^−1^ min^−1^) after 480 min. Thus, the OV-rich α-Fe_2_O_3_ NPs prepared at 900 °C are the most active sample for methane decomposition, and this is due to their possession of abundant oxygen vacancies, which serve as effective active sites for adsorption methane.

### 3.4. Proposed Reaction Mechanism

**Figure 15:**
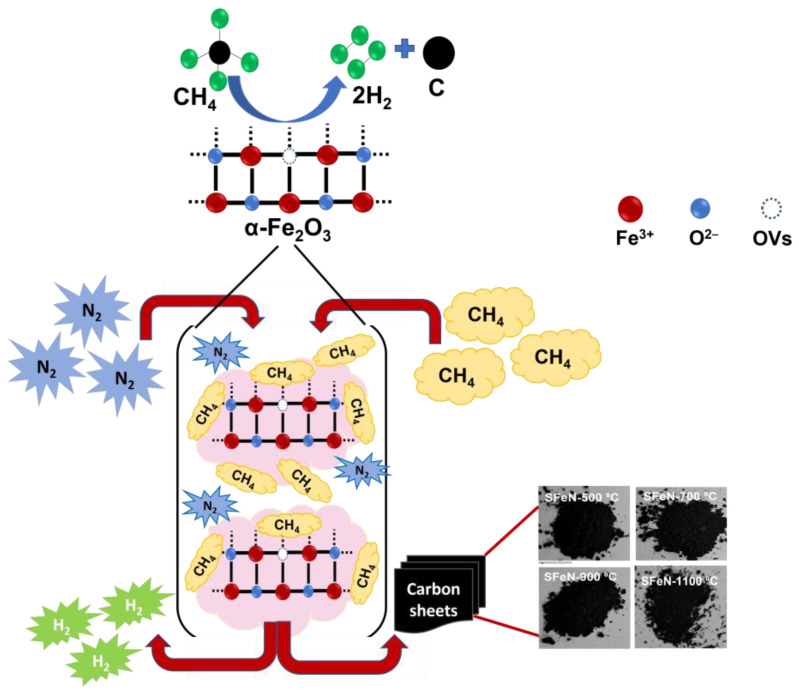
A schematic diagram depicting the proposed reaction mechanism of TCMD by the unsupported OV-rich α-Fe_2_O_3_ NPs.

Available evidence from previous studies indicates that the surface features of catalysts are the main drivers of catalytic methane decomposition. The XPS analysis performed as a part of this work revealed that the obtained OV-rich α-Fe_2_O_3_ NPs at calcination temperatures such as 900 °C possess abundant oxygen vacancies, which would, in turn, increase the number of stable and active sites for the adsorption of methane. It has also been established that the introduction of OVs into the bulk α-Fe_2_O_3_ material can weaken the Fe–O bond strength and improve the lattice oxygen mobility, thus catalyzing the reduction of iron oxide to form the active phase for methane decomposition. Therefore, it is reasonable to assume that methane adsorbs on the surface-active sites of the catalyst and reacts with the reducible species, e.g., free Fe_2_O_3_ clusters (adjacent Fe^3+^), surface oxygen species, and defects such as OVs, at the 800 °C reaction temperature. As a result, the C–H bonds on the catalyst surface would be gradually decomposed to generate H_2_ and nanocarbon materials. Thus, FeN-900 °C, which contains abundant oxygen vacancies, could play an important role in the enhancement of catalytic activity in the thermocatalytic decomposition of methane into hydrogen and nanocarbon materials. A schematic diagram depicting the proposed reaction mechanism of the OV-rich α-Fe_2_O_3_ NPs that are used for the thermocatalytic decomposition of methane is illustrated in Figure 15.

Furthermore, based on the results reported in extant studies [9,18,19], the reduction of α-Fe_2_O_3_ catalyst by CH_4_ occurs in three steps, namely Fe_2_O_3_ → Fe_3_O_4_ → FeO → Fe^0^. Once Fe^0^ is formed, it decomposes CH_4_, which results in the formation of Fe_3_C, a crucial step in the TCMD process for the formation of nanocarbon materials. The XRD, SEM, and XPS findings obtained in the current investigation indicate that the reduction of the OV-rich α-Fe_2_O_3_ catalysts follows the global mechanism (Fe_2_O_3_ → Fe^0^), whereby Fe_3_C is formed as a result of the reaction of Fe^0^ with the produced carbon from CH_4_ decomposition. Therefore, it is clear that Fe^0^ is an active phase for initializing the TCMD reaction at high temperatures (e.g., 800 °C), which suggests that the OV-rich α-Fe_2_O_3_ catalysts possess an effective catalytic nature for TCMD for hydrogen and nanocarbon production.

## 4. Conclusions

In this study, a scalable and cost-effective one-step approach was developed for obtaining efficient unsupported OV-rich α-Fe_2_O_3_ NPs with abundant oxygen vacancies on the surface via the thermal decomposition of ferric nitrate at different temperatures (500, 700, 900, and 1100 °C). The obtained OV-rich α-Fe_2_O_3_ NPs at different calcination temperatures were employed as catalysts for the thermocatalytic decomposition of methane to produce H_2_ and nanocarbon. Further investigations confirmed that the introduction of OVs on the as-prepared OV-rich α-Fe_2_O_3_ NPs affected the characteristics and thermocatalytic performance of these catalysts. Based on the XPS analysis, the OVs on the OV-rich α-Fe_2_O_3_ surface were responsible for the increase in the number of active sites, thus aiding methane absorption. The morphological characterization results revealed that the most commonly obtained spent catalysts were nanosheets, which were the predominant carbon form deposited on the spent catalysts. According to the XRD results, the TCMD reduction follows the Fe_2_O_3_ → Fe^0^ global mechanism. By evaluating the thermocatalytic activity of the obtained OV-rich α-Fe_2_O_3_ NPs in terms of TCMD for hydrogen production and nanocarbon formation, it was determined that 900 °C calcination temperature is optimal for producing highly efficient OV-rich α-Fe_2_O_3_ NPs, which showed the highest methane conversion (22.5%) and hydrogen production rate (47.0 × 10^−5^ mol H_2_ g^−1^ min^−1^) after 480 min. Such outstanding thermocatalytic performance of OV-rich α-Fe_2_O_3_ NPs is attributed to the presence of abundant OVs on their surfaces, which serve as effective methane adsorption sites.

## Figures and Tables

**Table 1:** Textural properties of the obtained OV-rich α-Fe_2_O_3_ NPs at different calcination temperatures for 5 h at ambient conditions.

Catalyst	Surface Area (m^2^/g)	Pore Volume (cm^3^/g)	Pore Radius (nm)
FeN-500 °C	16.7	0.34	19.1
FeN-700 °C	7.8	0.23	17.0
FeN-900 °C	5.5	0.04	15.3
FeN-1100 °C	3.9	0.04	21.6

**Table 2:** Literature review on Fe-based catalysts for TCMD.

Used Catalysts	Preparation Method	WHSV [L/(g_cat_∙h)]	TCMD Condition	Initial CH_4_ Conversion	Reaction Time (Min)	Final CH_4_ Conversion	Ref.
Fe–Al, 40% Fe	Fusion	7.5	CH_4_, 750 °C	66%	120	19%	[16]
Fe–Ce, 27% Fe	Co-precipitation	4.5	CH_4_, 800 °C	35%	360	49%	[40]
Fe–La, 27% Fe	Co-precipitation	4.5	CH_4_, 800 °C	24%	360	33%	[40]
Fe-Mg, 50% Fe	Chemical impregnation	1.5	CH_4_, 700 °C	5%	600	27%	[41]
Fe-Mo-Al, 62% Fe	Fusion	1.5	CH_4_, 750 °C	75%	180	70%	[42]
Tierga ore, 52.6% Fe	None	2	CH_4_, 800 °C	30%	180	32%	[43]
Ilmenite ore, 33.3% Fe	None	2	CH_4_, 800 °C	8%	180	10%	[43]
Unsupported α-Fe_2_O_3_	Calcination	2.4	CH_4_, 800 °C	35.2	480	22.5%	This work

## Data Availability

The data presented in this study are available on request from the corresponding author.
